# RNA polymerase II depletion from the inactive X chromosome territory is not mediated by physical compartmentalization

**DOI:** 10.1038/s41594-023-01008-5

**Published:** 2023-06-08

**Authors:** Samuel Collombet, Isabell Rall, Claire Dugast-Darzacq, Alec Heckert, Aliaksandr Halavatyi, Agnes Le Saux, Gina Dailey, Xavier Darzacq, Edith Heard

**Affiliations:** 1 European Molecular Biology Laboratory, Heidelberg, Germany; 2 Department of Molecular and Cell Biology, University of California, Berkeley, Berkeley, CA USA; 3 Li Ka Shing Center for Biomedical and Health Sciences, University of California, Berkeley, Berkeley, CA USA; 4 Curie Institute, PSL Research University, CNRS UMR3215, INSERM U934, UPMC Paris-Sorbonne, Paris, France; 5 College de France, Paris, France

**Keywords:** Nuclear organization, Single-molecule biophysics, Gene silencing, Microscopy, Transcription

## Abstract

Subnuclear compartmentalization has been proposed to play an important role in gene regulation by segregating active and inactive parts of the genome in distinct physical and biochemical environments. During X chromosome inactivation (XCI), the noncoding *Xist* RNA coats the X chromosome, triggers gene silencing and forms a dense body of heterochromatin from which the transcription machinery appears to be excluded. Phase separation has been proposed to be involved in XCI, and might explain the exclusion of the transcription machinery by preventing its diffusion into the *Xist*-coated territory. Here, using quantitative fluorescence microscopy and single-particle tracking, we show that RNA polymerase II (RNAPII) freely accesses the *Xist* territory during the initiation of XCI. Instead, the apparent depletion of RNAPII is due to the loss of its chromatin stably bound fraction. These findings indicate that initial exclusion of RNAPII from the inactive X reflects the absence of actively transcribing RNAPII, rather than a consequence of putative physical compartmentalization of the inactive X heterochromatin domain.

## Abstract (web summary)

The authors show that the apparent depletion of RNAPII from the inactive X chromosome territory is not due to a biophysical compartmentalization effect, but rather due to the loss of RNAPII bound fraction on chromatin.

## Main

In female eutherian mammals, one of the two X chromosomes becomes silenced through the process of XCI. This is controlled by the noncoding *Xist* RNA that becomes up-regulated from one of the two X chromosomes during early development, coating the chromosome *in cis* and triggering chromosome-wide gene silencing. One of the earliest events occurring during XCI is the very rapid exclusion of the transcriptional machinery from the *Xist*-coated part of the X chromosome territory, both in early embryos in vivo^1^ and in early differentiating mouse embryonic stem cells (mESCs)^2^. This has led to the suggestions that the Xi may form a specific subnuclear compartment that could constitute a biophysical ‘barrier’ preventing RNAPII and other transcription factors from entering the *Xist*-coated territory to ensure and/or to maintain gene silencing^3^. Indeed X-linked genes become relocated into the Xist compartment as they become silenced, while genes that escape XCI tend to remain located at the outside or at the periphery of the *Xist* RNA domain^2,4^, although whether this gene relocation is a cause or a consequence of X chromosome gene silencing events is still not clear.

*Xist* is a multitasking molecule that recruits many proteins via its conserved repeats to the future inactive X chromosome (Xi). This includes SPEN, which is brought via the *Xist* A repeats and is essential for gene silencing^5,6^. The PRC1 Polycomb complex is also recruited via *Xist* B repeats, while PRC2 gets accumulated on the Xi downstream of this^7–10^. Other factors are also recruited via *Xist* E repeats, including CIZ1 at the onset of XCI (ref. ^11^), and PTBP1, MATR3 and CELF1 at later stages^12^. These proteins become highly accumulated on the inactive X in a *Xist* dependent manner. Protein–RNA and protein–protein interactions have been proposed to allow their accumulations through liquid–liquid phase separation, forming so-called membraneless organelles such as the nucleolus or stress granules. A number of Xist-recruited factors, including SPEN, PRC1 and PTBP1-MATR3, form low affinity protein–protein interactions^12–14^, suggested to increase molecular crowding in the *Xist* compartment and participate in the compaction of chromatin^14^. Accordingly, phase separation has been proposed to underlie the compartmentalization of the inactive X^15^. This hypothesis would offer a simple mechanism for the exclusion of RNAPII, as *Xist* would trigger the formation of a heterochromatic membraneless organelle from which the transcription machinery is physically excluded. However, other mechanisms have been shown to allow apparent nuclear compartmentalization, such as modulation in protein–DNA binding rate^16^ or more complex models^17^. In other systems, while phase separation has been shown to occur, further investigation shows that it does not necessarily play a clear functional role, for example in the formation of constitutive heterochromatin^18^, or during transcriptional activation by enhancers and transcription factors^19,20^.

To investigate the nature of the *Xist* RNA nuclear compartment and its relationship to RNAPII, we have applied quantitative fluorescence microscopy and single-particle tracking (SPT) to assess the concentrations and the dynamics of RNAPII in living cells, both inside and outside the *Xist* compartment as it forms and gene silencing initiates.

## Results

### RNAPII concentration within the *Xist* RNA compartment

**Fig. 1:**
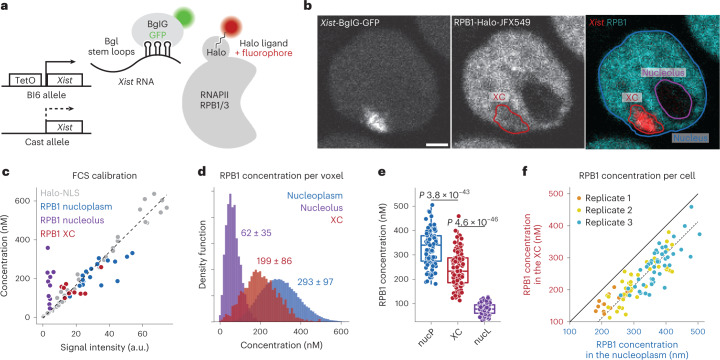
RNAPII concentration in the Xist compartment. **a**, Scheme of Xist and RNAPII tagging for combined live-cell imaging. **b**, Representative image (from 107 different single cells) of confocal microscopy of Xist (BglG-GFP) and RNAPII (RPB1-Halo) in live cells (single *z* stack) after 24 h of *Xist* induction (doxycycline treatment) with overlaid segmentation of nucleus, nucleoli and Xi (Methods). Scale bar, 2 μm. **c**, Calibration of signal intensity from point scanning imaging with FCS measured concentrations. Each dot represents a single measurement from a single cell. The linear calibration is established only on the freely diffusing Halo-NLS. Note that fluorescence intensity in the nucleolus was below the threshold of robust FCS measurement (Extended Data Fig. 2d), leading to artifactual concentration estimation. **d**, Calibrated RNAPII (RPB1) concentration per voxel for a single cell (cell shown in **b**), based on the calibration in **c**. The average concentration per region for this cell is indicated (±95% confidence interval). **e**, Distribution of average concentration per region per cell, for all cells after 24 h of Xist induction. Each dot represents a single cell (*n* = 107). *P* values of the differences are indicated on top (*t*-test two-sided, paired data). Boxplots represent the median (center) first and third quartile (hinges) and ±1.5 × IQR (whiskers). **f**, Average RPB1 concentration in the XC versus nucleoplasm. Each dot represents a single cell.

**Extended Data Fig. 1:**
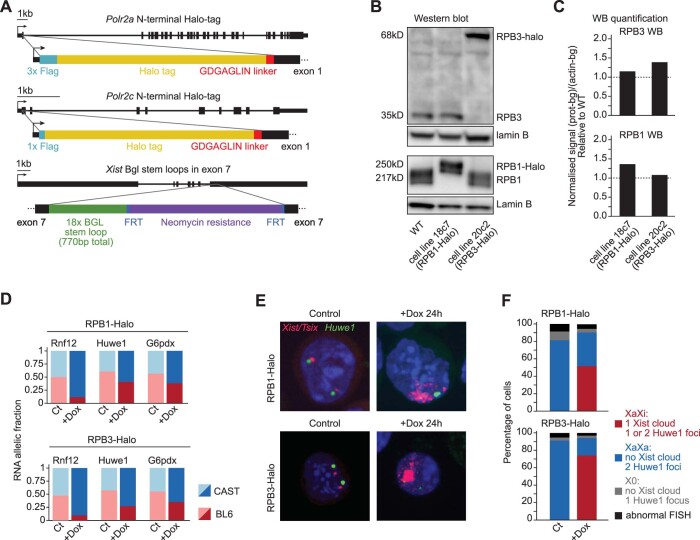
Cell line characterisation. **a** scheme of genetic engineering for RPB1-Halo, RPB3-Halo and Xist-Bgl. **b**. Western blot for RPB1 and RPB3 in untagged cell line, RPB1-Halo and RPB3-Halo. Whole image of the WB are shown in Source Data. **c**. Quantification of the westernblot signal from B. **d**. Allelic expression of Rnf12, Huwe1 and G6pdx measured by RNA pyrosequencing in cells before (Ct) and after 24 h Xist induction. **e**. Representative examples of RNA FISH for Xist and Huwe1 in cells before (Ct) and after 24 h Xist induction. **f**. Quantifications of the percentage of cells showing Xist induction (XaXi, red) no induction (XaXa, blue) or other phenotype before and after 24 h Xist induction. Source data

**Extended Data Fig. 2:**
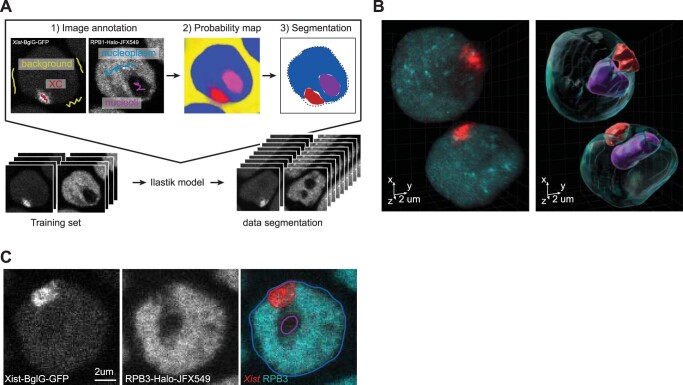
Live imaging of *Xist* RNA and RNAPII, segmentation and FCS-CI. **a** Summary of the 3D segmentation workflow using Ilastik^24^. **b** 3D rendering of *Xist*-BglG-GFP and RPB1-Halo signals in live-cell confocal imaging; and of the nucleoplasm, XC and nucleolus segmentation. **c** Representative image (from 92 single cells) of confocal microscopy of *Xist*-BglG-GFP and RPB3-Halo in live cells (single Z stack) after 24 h of Xist induction (doxycycline treatment) with overlaid segmentation of nucleus, nucleoli and Xi (see Methods).

To assess the dynamics and nature of the RNAPII-depleted *Xist* RNA compartment, we took advantage of a mouse embryonic stem cell line (mESC) in which the endogenous *Xist* gene on one chromosome is controlled by a doxycycline inducible promoter^21^. This allows for rapid and synchronous induction of *Xist* expression and X chromosome inactivation (XCI). To visualize *Xist* RNA and RNAPII in live cells, we tagged *Xist* (on the doxycycline inducible allele) with BGL stem loops that can be specifically bound by a BglG protein fused to a green fluorescent protein (GFP)^6,22^. In the same cells, we generated homozygous Halo Tag knock-ins at the endogenous RNA Polr2a and Polr2c genes, encoding the RPB1 and RPB3 proteins respectively, which are two major subunits of RNAPII (Fig. 1a and Extended Data Fig. 1a,b). Both the RPB1- and RPB3-Halo knock-in cell lines displayed slightly higher levels of the tagged proteins compared to wild-type cells (Extended Data Fig. 1c) possibly due to a stabilization of the protein by the tag. After 24 h *Xist* induction, which has been previously shown to be sufficient for global silencing of almost all X-linked genes^6,23^, cells show efficient *Xist* RNA coating (in more than 50% of cells) and X-linked gene silencing (Extended Data Fig. 1d–f). Using confocal microscopy, we imaged *Xist*-Bgl-GFP and RNAPII-Halo in three-dimensions (with 0.48 μm between stacks, Methods) in live cells following 24 h of *Xist* induction, and systematically segmented the *Xist* compartment (XC), the nucleoplasm and nucleoli (as control regions where RNAPII is excluded) using robust machine learning segmentation tools^24^ (Fig. 1b and Extended Data Fig. 2a–c).

**Extended Data Fig. 3:**
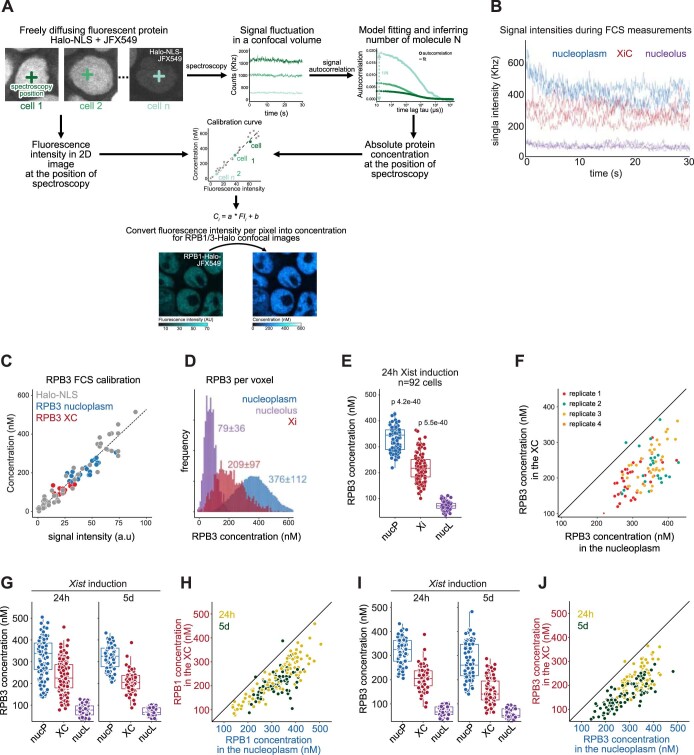
FCS Calibrated imaging. **a**. FCS-CI workflow. **b**. Representative example of signal intensities and fluctuation during FCS measurement in the nucleoplasm, XC and nucleolus. **c**. Calibration of RPB3 signal intensity from point scanning imaging with FCS measured concentrations. Each dot represents a single measurement from a single cell. The linear calibration is established only on the freely diffusing Halo-NLS. **d**. Calibrated RPB3 concentration per voxel for the nucleus shown in C, based on the calibration in E. the average concentration per region is indicated (± 95% confidence interval). **e**. distribution of RPB3 average concentration per region per cell after 24 h of Xist induction. Each dot represents a single cell (n = 92). P-values of the differences are indicated on top (*t*-test two sided, paired data). *Boxplots represent the median (center) 1*^*st*^ *and 3*^*rd*^ quartile (hinges) and +/− 1.5*IQR (whiskers). **f**. RPB3 Concentration in the XC versus nucleoplasm. Each dot represents a single cell. **(g)** Distribution of average RPB1 concentration per region per cell, for all cells after Xist induction for 24 h (left) and 5 days (right). Each dot represents a single cell. **(h)** Average RPB1 Concentration in the XC versus nucleoplasm, colour by time of treatment (24 h in yellow, 5 days in green). Each dot represents a single cell. *Boxplots represent the median (center) 1*^*st*^ *and 3*^*rd*^ quartile (hinges) and +/− 1.5*IQR (whiskers). **(i)** and **(j)** are the same as (G) and (H) for RPB3. *Boxplots represent the median (center) 1*^*st*^ *and 3*^*rd*^ quartile (hinges) and +/− 1.5*IQR (whiskers).

We then assessed whether RNAPII was completely excluded from the *Xist* RNA territory or whether it could still be detected within the *Xist* domain. We used fluorescence correlation spectroscopy with calibrated imaging (FCS–CI)^25^, where FCS measurements on a freely diffusing control (Halo-NLS) allow calibrating fluorescence intensity from confocal images into absolute concentration (Fig. 1c and Extended Data Fig. 3a,b). FCS measurements for RNAPII either inside the *Xist* RNA territory or in the nucleoplasm, followed the same linear trend as the freely diffusing Halo-NLS control (Fig. 1c and Extended Data Fig. 3c), indicating that RNAPII concentration in these regions could be robustly measured. We then used this calibration curve to convert three-dimensional (3D) image voxel intensity to local concentration (Fig. 1d and Extended Data Fig. 3d) and found that the concentrations of RPB1 were significantly lower in the XC compared to the nucleoplasm (Fig. 1e), with an average reduction of 88 nM. RPB1 levels nevertheless remain substantial within the *Xist* territory, with an average concentration of 207 nM (corresponding to an average 1,165 molecules of RPB1 per XC). Similar results were found for RPB3 with an average concentration of 220 nM (average of 934 molecules per XC) (Extended Data Fig. 3e). The concentration of both RPB1 and RPB3 in the *Xist* compartment scaled linearly with their nucleoplasmic concentration (Fig. 1f and Extended Data Fig. 3f) showing a constant offset. This suggests that RNAPII concentration is at an equilibrium between the nucleoplasm and the XC and that its diffusion into the Xist territory is not hindered. We have previously shown that virtually all X-linked genes are silenced after 24 h *Xist* induction^6,23^. To determine whether the loss of RNAPII we observed after 24 h was maximal and that no further exclusion occurs with longer *Xist* induction times, we induced *Xist* expression for 5 days, and found similar reductions in RPB1 and RPB3 concentrations (Extended Data Fig. 3g–j). This is consistent with previous results showing that RNAPII depletion from the XC is one of the earliest events during XCI initiation in vivo (1) and in vitro (2). Altogether, these results indicate that gene silencing on the inactive X is not mediated by a physical exclusion of RNAPII from the *Xist* territory.

### RNAPII can diffuse freely through the *Xist* compartment

**Fig. 2:**
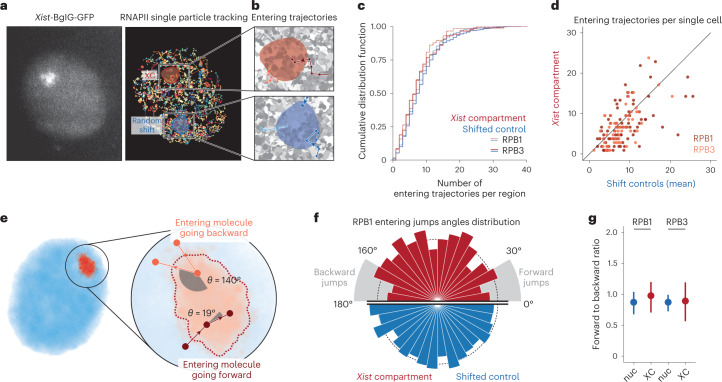
Characterization of RNAPII flux with XC. **a**, Example of *Xist*-BGL-GFP imaging and RNAPII RPB1 SPT (5.477 ms between frame, 1 ms exposure, 3 min tracking) in the same cell. Each trajectory is shown with random colors. The red area shows the segmentation of the *Xist* compartment, and the blue area a random spatial shift of this region (Methods). **b**, Enlargement of the XC region and random shift to highlight entering trajectories of RNAPII into the XC. **c**, Cumulative distribution function of the number of entering trajectories in the XC (red) and random shifts (blue) for RPB1 (plain line) and RPB3 (dashed line). Only trajectories with a mean square root displacement (MSRD) > 200 nm were selected to ensure using only freely diffusing molecules (Methods). **d**, Scatter plot of the number of trajectories entering the XC (*y* axis) and the average number of trajectories entering the shift controls (*x* axis) in the same single cell, for RPB1 (red) and RPB3 (orange). **e**, If a molecule ‘bounces back’, its trajectory should display large angles between jumps, while molecules that ‘move forward’ should display small angles. Molecules in Brownian motion in free space should show no preference. **f**, Distributions of entering trajectories angles between the entering jump and the following one (as depicted in **e**) for RPB1 trajectories entering the XC (red, *n* = 556 entering jumps) or shifted control regions (blue, *n* = 4,408 entering jumps). The radius of the bar represents the density of counts. **g**, Ratio of forward angles (0 to 30°)/backward angles (160 to 180°) as previously done^27,28^. The dot represents the fraction estimated from all pooled trajectories (*n* = 556/4,408 and 346/3,310 trajectories entering XC/shift control region, for RBP1 and RPB3, respectively), and the error bar represents the standard deviation (centered on the mean estimated value) from 50 bootstrap subsampling of *n* = 250 entering jumps (Methods).

**Extended Data Fig. 4:**
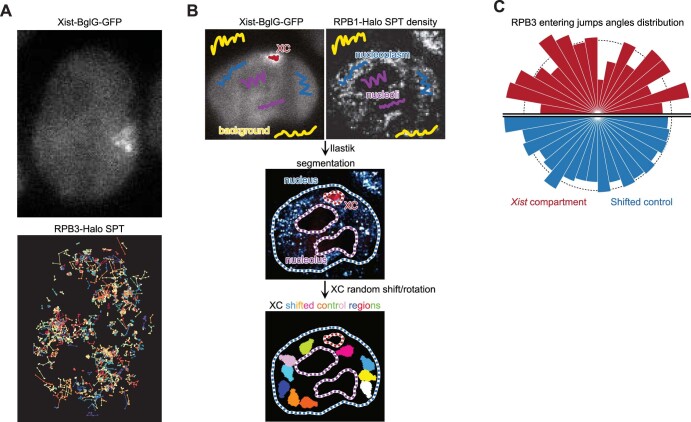
Single particle tracking data analysis. **a**. Representative example of RPB3-Halo single particle tracking after 24 h *Xist* induction. **b**. workflow of the SPT data segmentation and XC shifted control regions. **c**. distribution of jump angles for RBP3 jump entering the XC.

We next more directly addressed whether the reduction in RNAPII concentration on the XC was due to a ‘barrier effect’ reducing RNAPII flux exchanging in and out of the domain. We performed SPT using a photo-activatable Halo-PA-JF646, and high-speed imaging (5.477 ms frame interval and 1 ms excitation pulse) to capture the trajectories of diffusing molecules (Fig. 2a and Extended Data Fig. 4a)^26,27^. We then compared the flux of RNAPII molecules entering the Xi territory to comparable regions in the nucleoplasm (Fig. 2b, Extended Data Fig. 4b and Methods). XC territories have different sizes and shapes, and we first compared the distribution of entering events and found no significant difference for both RPB1 and RPB3 (Kolmogorov–Smirnov test *P* values of 0.41 and 0.56, respectively) (Fig. 2c). At the single cell level, RNAPII flux is strongly correlated with that in the rest of the nucleoplasm (Fig. 2d). The observed differences in concentration (Fig. 1f) cannot therefore be attributed to a potential domain exclusion mechanism such as phase separation. Finally, we wondered whether RNAPII molecules entering the XiC are more likely to return to the nucleoplasm (Fig. 2e). Looking at the statistical distribution of angles of entering trajectories, we did not observe a clear difference inside and outside XiC, neither for RPB1 nor RPB3 (Fig. 2f and Extended Data Fig. 4c). Quantifying the ratio of forward (0 to 30°) and backward angles (160 to 180°) as previously done^27,28^, we found a small increase in forward angles in the XiC compared to shifted regions for RPB1 and no differences for RPB3 (Fig. 2g), suggesting that the trajectories of RNAPII molecules entering the XiC are not significantly affected and do not tend to diffuse backward in the nucleoplasm. Of note, we observed a lower proportion of forward and backward jumps (relative to lateral ones, that is angles between 30° and 160°) inside the XC compared to the nucleoplasm. This could be explained by a difference in the proportion of bound and free molecules.

Altogether, these results show that RNAPII can freely enter the *Xist* compartment.

### RNAPII diffusion is not altered within the *Xist* territory

**Fig. 3:**
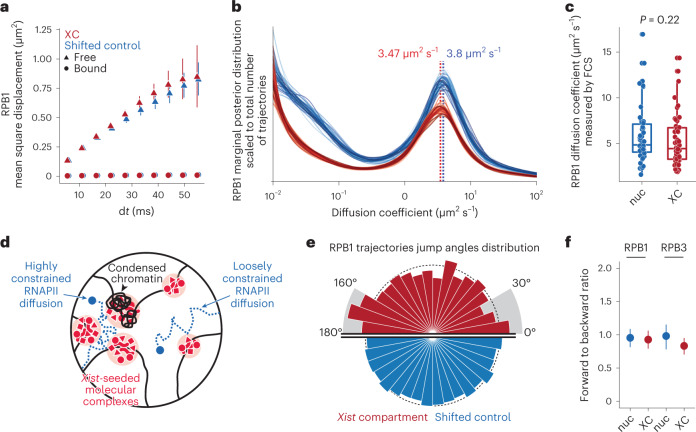
RNAPII diffusion on the Xi. **a**, MSD at increasing time interval dt for RPB1 SPT trajectories inside *Xist* compartment (red) or in shifted control regions (blue, Fig. 2b). Trajectories are split into free and bound based on their average jump length (MSRD > 200 nm for free, and <100 nm for bound), as in Fig. 2. The dots and error bars represent the mean and standard deviation of 50 bootstraps subsampling of 3,000 trajectories (Methods). **b**, Distribution of diffusion coefficient inferred using spagl (Methods) for RPB1 SPT trajectories inside the Xist compartment and in shifted control regions. The marginal posterior distribution is scaled to the average number of trajectories in Xist compartment and in shifted control regions. **c**, Diffusion coefficients of RPB1 free fraction based on FCS measurement inside the Xist compartment and in the nucleoplasm, and fitting a two-component model (bound and free, Methods). Each dot represents a single measurement in a single cell (*n* = 53). The indicated *P* value is calculated with a *t*-test (two-sided, paired data). Boxplots represent the median (center) first and third quartiles (hinges) and *±*1.5 × IQR (whiskers). **d**, Schematic representing how different environments might constrain RNAPII (in blue) diffusion. On the left side, *Xist*-seeded molecular complexes (in red) and dense chromatin (black) occupy a significant space that constrains RNAPII diffusion. On the right side, protein complexes and chromatin do not occupy a significantly higher space and RNAPII diffusion is not affected. **e**, Distributions of jump angles for RPB1 trajectories entering the XC (red, top) or control regions (blue, bottom). The radius of the bar represents the density of counts. **f**, Ratio of forward angles (0 to 30°)/backward angles (150 to 180°). The dots represent the fraction estimated from all free trajectories (MSRD > 200 nm, *n* = 1410/13,335 and 834/8,826 trajectories in XC/shift, for RBP1 and RPB3, respectively), and the error bars represent the standard deviation (centered on the mean estimated value) from 50 bootstrap subsampling of 500 free trajectories (Methods).

**Extended Data Fig. 5:**
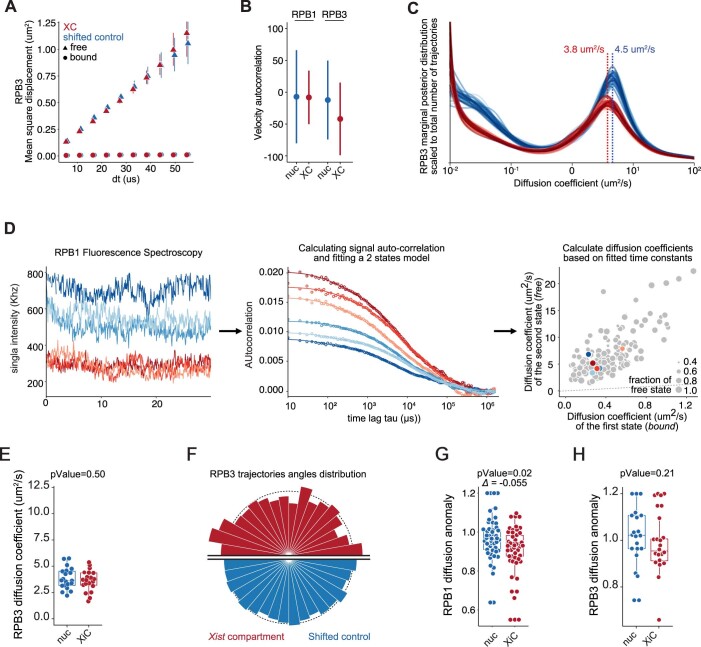
Characterisation of RNAPII diffusion on the Xi. **a**. Mean square displacement (MSD) at increasing time interval dt for RPB3 SPT trajectories inside Xist compartment (red) or in shifted control regions (blue, see Extended Data Fig. 4b). Trajectories are splitted into free and bound based on their average jump length (MSRD > 200 nm for free, and <100 nm for bound), as in Fig. 2. The dot and error bar represent the mean and standard deviation of 50 bootstraps subsampling of 3000 trajectories (see Methods). **b**. Velocity Auto-Correlation (VAC) for RPB1/3 SPT trajectories inside the *Xist* compartment (red) or in shifted control regions. The dot and error bar represent the mean and standard deviation of 50 bootstraps subsampling of 3000 trajectories (see Methods). **c**. Distribution of diffusion coefficient inferred using spagl (see Methods) for RPB1 SPT trajectories inside the Xist compartment and in shifted control regions. The marginal posterior distribution is scaled to the average number of trajectories in Xist compartment and in shifted control regions. **d. e**. Diffusion coefficients of RPB3 free fraction based on FCS measurement inside the Xist compartment and in the nucleoplasm, and fitting a two component model (bound and free, see Methods). Each dot represents a single measurement in a single cell (n = 20). The indicated P value is calculated with a *t*-test (two sided, paired data). *Boxplots represent the median (center) 1*^*st*^ *and 3*^*rd*^ quartile (hinges) and +/− 1.5*IQR (whiskers). **f. g**. RPB1 diffusion anomaly exponent from FCS measurement inside and outside XC. Each dot represents a single cell (n = 53). *The indicated P-value is calculated with a t-test (two sided, paired data). Boxplots represent the median (center) 1*^*st*^ *and 3*^*rd*^ quartile (hinges) and +/− 1.5*IQR (whiskers). **h**. Same as G for RPB3 (n = 20). *The indicated P-value is calculated with a t-test (two sided, paired data). Boxplots represent the median (center) 1*^*st*^ *and 3*^*rd*^ quartile (hinges) and +/− 1.5*IQR (whiskers).

We then investigated whether the diffusion of RNAPII was affected inside a *Xist* territory. The mean square displacement (MSD) and velocity autocorrelation (VAC) are classical metrics of single molecule diffusion properties. We found a slight increase in MSD in the XC for RPB1 (Fig. 3a) and to a lesser extent for RPB3 (Extended Data Fig. 5a), and a reduction in VAC for RPB3 only (Extended Data Fig. 5b). However, both MSD and VAC estimations from fast SPT data have been reported to be subject to various biases due to the short length of trajectories and localization error^26,29,30^, and do not discriminate between different subpopulations. We have recently developed an approach to infer the distribution of diffusion coefficients from short SPT without a priori assumptions on a discrete number of states^30^. Looking at RPB1 and RPB3 in the nucleoplasm, we found two main populations: the first one with diffusion coefficients below 0.1 μm^2^ s^−1^ (Fig. 3b and Extended Data Fig. 5c), similar to values reported for histones and DNA locus tracking^26,30^ and therefore corresponding to the bound RNAPII molecules, and a second population with diffusion coefficient centered around 4 μm^2^ s^−1^ corresponding to freely diffusing molecules. Turning to trajectories on the *Xist* compartment, We observed a loss of the bound fraction, in particular for the molecules with diffusion coefficients between 0.02 and 0.1 μm^2^ s^−1^. On the other hand, the diffusion coefficient of the free fraction was virtually the same for RPB1 (3.47 on the XC and 3.8 in the nucleoplasm) and slightly reduced for RPB3 (3.8 versus 4.5). To confirm these results with another approach, we fitted a two components model to FCS measurements (Methods and Extended Data Fig. 5d) for RPB1 and RPB3, and found no significant differences in diffusion coefficients of the free fraction for both proteins (Fig. 3c and Extended Data Fig. 5e). Together, these results indicate no or minimal changes on RNAPII diffusion inside the *Xist* compartment.

The inactive X has previously been shown to be more compact than its active counterpart (roughly 1.2-fold compaction)^31,32^. In addition, *Xist*-recruited proteins such as SPEN, Ciz1 and PRC1 have recently been shown to form supra-molecular complexes on their recruitment on the inactive X^14^. Increased molecular crowding, due to protein complexes and/or higher chromatin density, could constrain the diffusion of RNAPII (Fig. 3d). Looking at the jump angle distribution for free RNAPII (MSRD > 200 nm), we did not observe any difference for RPB1 trajectories inside XC or in shifted control region (Fig. 3e,f), and only a slightly lower forward/backward ratio for RPB3 (0.83 in XC versus 0.97 in shifted control region, Fig. 3f and Extended Data Fig. 5f). This result indicates that molecular crowding on the *Xist* territory has only a minor effect (if any) on RNAPII diffusion. Accordingly, we found only a slight reduction in diffusion anomaly, lower values indicating diffusion constraints from a more crowded environment, inferred by FCS measurement for RPB1 (Extended Data Fig. 5g) and no differences for RPB3 (Extended Data Fig. 5h).

Together, these results show that neither chromatin compaction nor *Xist* RNA associated molecular complexes affect RNAPII diffusion during XCI initiation.

### Loss of the RNAPII stably bound fraction

**Fig. 4:**
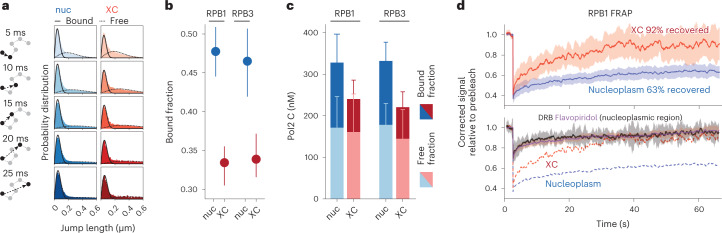
RNAPII dynamics on XC. **a**, Fitting of a two-component model (bound versus freely diffusing molecules) to the distribution of jump length (that is, distance between localization *n* and *n* + d*t* from the same trajectory) at different time scales, for trajectories inside the XC or outside (nuc). All data from 96 single cells are pooled. **b**, Estimates of the bound fraction inside and outside the XC, for RPB1 and RPB3. The dots represent the fraction estimated from all pooled trajectories, and the error bars the standard deviation (centered on the mean estimated value) from 50 bootstrap subsampling of 3,000 trajectories (Methods). **c**, Concentration of RPB1 and RPB3 (from Fig. 1f and Extended Data Fig. 2g) scaled by the bound and free fraction from **b**. The error bars represent the 95% confidence interval for the product of concentration and bound/free fraction (centered on the product value) and is calculated using the delta method^45^ (Methods). **d**, FRAP experiment for the XC (red) or control nucleoplasmic region (blue) in the top panel, and in nucleoplasmic regions after treatment with DRB (black) or Flavopiridol (purple) in the bottom panel. Signal is normalized to the signal before bleaching (Methods). The line represents the mean of signals (*n* = 15 cells) and the shade its 95% confidence interval. In the bottom panel, the dotted lines represent the mean of signal for XC and nucleoplasmic regions in untreated cells and are the same as the plain lines in the top panel.

**Extended Data Fig. 6:**
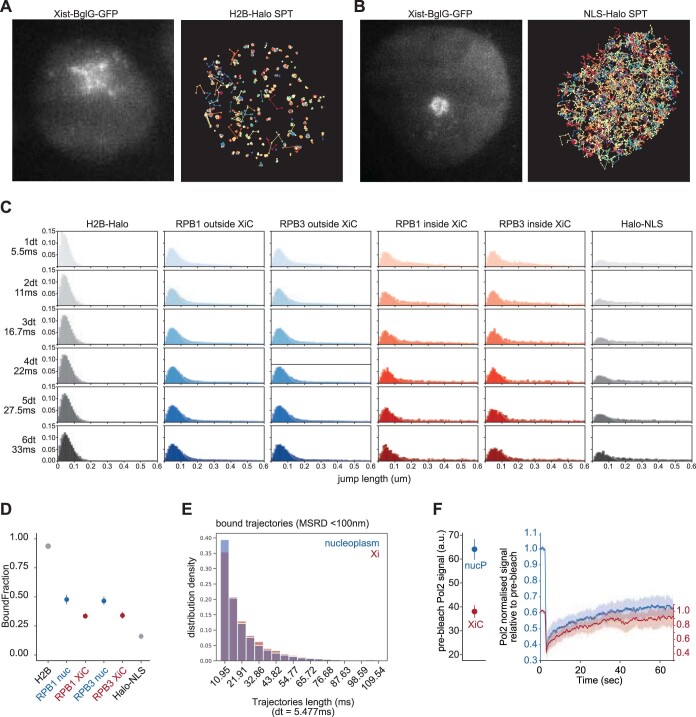
Single particle tracking and FRAP. **a**. Representative example of histone H2B-Halo single particle tracking after 24 h *Xist* induction. **b**. Representative example of Nls-Halo single particle tracking after 24 h *Xist* induction. **c**. Distribution of jump length in single particle tracking RPB1, RPB3 and the ‘bound’ histone H2B-Halo and ‘free’ Halo-NLS controls, and **d**. Estimated bound fractions. The dot represents the fraction estimated from all pooled trajectories, and the error bar the standard deviation *(centered on the mean estimated value)* from 50 bootstrap subsampling of 3,000 trajectories (see Methods). **e**. Distribution of tracking duration for bound RPB1 molecules (MSRD < 100 nm) inside and outside XC. **f**. Fluorescence recovery after photobleaching (FRAP) signal as in Fig. 3d, but where the signal in XC is scaled to its prebleached intensity relative to the nucleoplasmic signal. Left panel: *the dots represent the mean of signals (n* = *15 cells) and the error bars the 95% confidence interval. Right panel: the line represents the mean of signals (n* = *15 cells) and the shade its 95% confidence interval*.

Given these results, we hypothesized that the apparent decrease in RNAPII concentration (Fig. 1e) could be the result of a loss of the bound fraction of RNAPII on the *Xist* RNA coated inactive X chromosome, while the diffusing fraction is at an equilibrium with the nucleoplasm. This hypothesis would also explain the constant offset in RNAPII concentration on the XC independently of the nucleoplasmic concentration (Fig. 1f): assuming RNAPII binding sites on DNA are saturated over the range of global RNAPII concentrations we observed, the inhibition of RNAPII binding to its target sites on the inactive X would result in a constant loss of bound fraction in each cell. Fitting a two components model (bound versus freely diffusing) to our SPT data (Fig. 4a and Extended Data Fig. 6a–c), we found a significant reduction in the bound fraction on the XC for both RPB1 and RPB3 (Fig. 4b). Scaling the concentration measured by FCS–CI (from Fig. 1f) by the bound and/or free fraction measured by SPT (Fig. 4c), we confirmed that the amount of freely diffusing RNAPII is not significantly different between the XC and the nucleoplasm (in agreement with no impairment in RNAPII diffusion) but the amount of bound RNAPII is reduced. Yet approximately one-third of RNAPII molecules on XC are bound, presumably specifically immobilized on chromatin (Extended Data Fig. 6d). Given that fast SPT is not ideal to interrogate long binding events as observed with elongating RNAPII in the nucleoplasm (Extended Data Fig. 6e)^26^ we investigated further this bound fraction using fluorescence recovery after photobleaching (FRAP). Using FRAP we found that almost all fluorescence on the XC was recovered 1 min after photobleaching (Fig. 4d, top panel) while only 63% was recovered on a region of the same size in the nucleoplasm, showing that the 33% ‘bound’ fraction on XC revealed by SPT corresponds to RNAPII molecules only transiently immobile, and that the ‘stably’ bound fraction (approximately 40% of RNAPII in the nucleoplasm) is completely lost. Scaling the FRAP signal in the XC to the nucleoplasmic level showed that similar absolute levels are recovered in both regions, corresponding to the initial levels of RNAPII on the XC (Extended Data Fig. 6f). This indicates that virtually all RNAPII on the XC correspond to the same amount of freely diffusing and transiently bound RNAPII as in the nucleoplasm. It has previously been shown that the inhibition of RNAPII transcription, either by blocking elongation or the formation of the pre-initiation complex, leads to the loss of RNAPII stably bound fraction^28,33^. The depletion of RNAPII observed on the XC would therefore be consistent with a blockage of transcription. We compared the dynamics of RNAPII on the XC, and in the nucleoplasm on chemical inhibition of elongation using d-ribofuranosylbenzimidazole (DRB) and flavopiridol^34^. Both compounds are inhibitors of the CDK9 kinase, part of the P-TEFb complex and which phosphorylate the Serine 2 of RPB1 C-terminal domain allowing entering into transcription elongation^34^. Measuring RNAPII stability by FRAP, both inhibitors led to a complete loss of the stably bound fraction in the nucleoplasm, similar to the dynamics observed inside the *Xist* compartment (Fig. 4d, lower panel). RNAPII signal recovery on the XC was slightly delayed, indicating slightly longer transient binding events compared to both DRB and flavopiridol treatment. These experiments indicate that the loss of RNAPII stably bound fraction on the *Xist* compartment would be consistent with a loss of the elongating RNAPII.

## Discussion

**Fig. 5:**
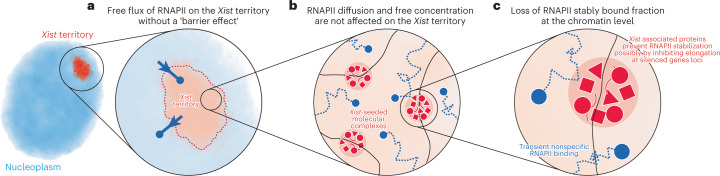
Summary of RNAPII dynamics on the *Xist* territory at multiple scales. RNAPII can freely diffuse through the *Xist*-coated territory during XCI, but loses its stable bound fraction on chromatin.

The sequestering functions of molecular compartmentalization in the nucleus have been long proposed to play a role in the regulation of gene expression. Local modifications in the concentrations of regulatory proteins, through biochemical or physical compartmentalization, may affect the efficiency of the processes they regulate. While this seems to be the case for repressors recruited by *Xist*, such as SPEN, PTBP1 and MATR3 (refs. ^12–14^), we show here that RNAPII diffusion is not prevented at the level of the all *Xist*-coated territory, resulting in a similar concentration of free RNAPII to that in the nucleoplasm (Fig. 5a). The inactive X chromosome has previously been shown to be a heterogeneous structure^35^, formed of distinct, tightly packed heterochromatin domains^36^. In addition, *Xist*-RNA associated proteins have been shown to form supra-molecular complexes through protein–protein interactions, which was proposed to increase molecular crowding and participate in chromatin compaction^12–14^. Phase separated protein domains are seen as regions where multivalent protein–protein interactions can modulate mass action, tweaking the ‘on rates’ of transcription factors binding to DNA^37^. Here we show that the nature of the multivalent assemblies decorating Xi chromatin during initiation of XCI does not modulate RNAPII ‘on rate’ by reducing the concentration of free RNAPII molecules. The fact that RNAPII diffusion coefficient and/or geometry and its transiently bound fraction are similar inside and outside the XC also suggest that the RNAPII target search process is not significantly affected inside the *Xist* RNA territory (Fig. 5b). Rather, *Xist* and its cofactors may act simply by preventing RNAPII binding to its DNA binding sites on chromatin, resulting in a loss of the stable bound fraction on the *Xist* territory (Fig. 5c). Taken together, our results indicate that the exclusion of RNAPII from the inactive X chromosome is not caused by a homogenous biophysical compartment-sequestering function, but rather by a modification of its chromatin that protects/represses RNAPII binding events. This is in agreement with the fact that the multivalent protein complexes involved in X inactivation decorate small chromatin domains in a manner not compatible with the formation of a large phase separated whole chromosome domains^14^.

Two mechanisms can be proposed to explain how *Xist*-associated factors prevent stable binding of the RNAPII: (1) they mask its binding sites on chromatin and impede RNAPII access, or (2) they prevent RNAPII stabilization at its target sites. Our FRAP data show that the amount of transiently bound RNAPII is the same inside and outside the XC (Extended Data Fig. 6f), therefore suggesting that *Xist* and its associated factors do not significantly prevent RNAPII access to DNA, but only its stabilization. In addition, the acute depletion of SPEN or the deletion of *Xist* A repeats (that are required for SPEN recruitment)^6,38^ result in a near complete loss of gene silencing, but do not affect *Xist* global accumulation on the X chromosome. However, deletion of the *Xist* A repeats prevents its coating of transcriptionally active regions of the genome^39^, suggesting that chromosomal *Xist* coating and the formation of supra-molecular complexes are not necessary for the initiation of gene silencing. Further experiments using live, super-resolution microscopy at the scale of a gene locus would be needed to confirm whether RNAPIi can access its target sites even transiently or whether *Xist* and its partners do have a physical, steric effect on RNAPII. Overall, these results indicate that the mechanism of gene silencing on the inactive X does not involve a mechanism of biophysical compartmentalization, but rather relies the biochemical modulation of transcription initiation.

We have recently shown that the initiation of gene silencing is mediated by the protein SPEN through its Spen paralogue and ortholog C-terminal (SPOC) domain^6^. While SPEN further allows the activation of HDAC3 to remove the active histone mark H3K27ac (refs. ^5,40^), the initial repression of transcription and the de-acetylation of histones happens through different pathways^6^. SPEN is recruited to the future inactive X chromosome as soon as *Xist* RNA accumulates, and becomes enriched at the promoters and enhancers of actively transcribed genes^6^. RNAPII was also identified in the interactome of SPEN’s SPOC domain^6^, suggesting that SPEN may interact with RNAPII. The SPOC domain of another protein (PHF3) has recently been shown to interact directly with RNAPII, allowing the repression of specific genes during neuronal differentiation^41^. Based on this, one can speculate that SPEN may directly inhibit transcription elongation by interacting with RNAPII, leading to its disengagement from chromatin and ultimately its apparent depletion on the *Xist*-coated territory. This hypothesis would also be consistent with the similar dynamics we observed by FRAP for RNAPII on the *Xist* territory and in the nucleoplasm on inhibition of elongation by DRB and flavopiridol.

The depletion of RNAPII within the *Xist* territory, which was described as one of the first events during XCI (refs. ^1,2^), is still observed on induction of a *Xist* mutant lacking its A repeats and therefore unable to silence genes^2^. This can be explained by the fact that in the absence of the A-repeat, as *Xist* cannot spread fully over active regions of the X chromosome^39^, these remain at the periphery of or outside the *Xist* territory^2^. In this case the *Xist* compartment consists of only silent regions of the chromosome, including most repetitive sequences^4^. Here, RNAPII is not expected to bind stably, and this therefore results in a reduction in concentration within the *Xist* domain, as visualized by RNAPII immunofluorescence or live imaging, or by Cot-1 RNA–fluorescence in situ hybridization (FISH).

Other transcription factors, including TBP and TAF10, have also been shown to be rapidly excluded from the *Xist RNA*-coated territory^2^. While we cannot conclude whether these factors might be subject to physical effects, the fact that a ‘inert probe’ such as our Halo-NLS does not seems to be excluded at all from the XC (Extended Data Fig. 6b) would go against a general physical effect for either small proteins such as a Halo-NLS or large complexes such as RNAPII.

The conclusions we have drawn here focus only on the initial steps of XCI, when *Xist* RNA first coats the X chromosome and gene silencing begins. Once *Xist* has triggered XCI, other factors and chromatin modifications accumulate on the inactive X, in some cases late during differentiation. For example, the PTBP1-MATR3 complex only accumulates on the Xi at around day 4 of differentiation^12^). While gene silencing is almost complete after 24 h of *Xist* induction in ESCs, some genes remain expressed at low levels and should therefore retain some stably bound, elongating RNAPII. The fact that we see virtually no stably bound RNAPII on the Xi by FRAP suggests that either nonfully silenced genes are located at the periphery of the XC territory when being expressed, or that the RNAPII molecules bound to those genes represent a negligible fraction compare to the one freely diffusing in the XC. While live tracking of RNAPII single molecule and DNA loci remains challenging, it will be interesting in the future to investigate when and where can RNAPII bind to these expressed loci on the Xi and how this dynamic relates to RNA synthesis. Furthermore, the inactive X chromosome has been shown to occupy a smaller volume (roughly 1.2-fold) than its active counterpart in differentiated cells in culture and in vivo blastocyst stage embryos^31,32,42^. Indeed, recent reports suggest that this compaction occurs only after several days of differentiation^14^, probably through the late recruitment of the SMCHD1 protein^43,44^. These factors may affect RNAPII diffusion and lead to the formation of a biophysical compartment, at these later stages of differentiation. Therefore, while our results show that impeded RNAPII diffusion within the *Xist*-coated domain of the X is not required for the initiation of gene silencing, it could still play a later role in the maintenance of XCI.

## Methods

### Cell culture and treatment

Mouse XX ESCs (TX1072) were grown on 0.1% gelatin-coated flasks in 8% CO_2_ at 37 °C and cultured in mESC media serum + 2i + leukemia inhibitory factor (LIF): DMEM (Sigma) without phenol-red, 15% FBS (Gibco), l-glutamin (584 mg l^−1^), nonessential amino acids (ThermoFisher no. 15140122, 6 ml l^−1^), sodium pyruvate (110 mg l^−1^), 0.1 mM β-mercaptoethanol, 1,000 U ml^−1^ LIF (Merck ESG110), CHIR99021 (3 μM) and PD0325901 (1 μM). *Xist* expression was induced on administration of doxycycline (2 μg ml^−1^).

### CRISPR–Cas9 genome editing

#### Transfection

All transgenic insertions were performed using the 4D nucleofector system from Lonza. For each nucleofection, 5 million cells were electroporated with in presence of the plasmids (MidiPreps). For targeted knock-in (RBP1- and RPB3-Halo, BglG-GFP at Tigre), 2.5 μg of nonlinearized targeting vectors and 2.5 μg of single-guide RNA were used. For Halo-NLS and H2b-Halo, 2.5 μg of piggybac construct containing the transgene and 2.5 μg of transposase plasmid were used.

#### Selection

For BglG-GFP knock-in at Tigre, the insert also contained a puromycin selection cassette. After nucleofection, cells were split into 10 cm with serial dilution (1/10, 1/100 and 1/1,000). Then 48 h after cell seeding, puromycin was added to culture media (0.4 μg ml^−1^). After 1 week of selection, single clonal colonies were picked (Clonal expansion).

#### FACS sorting

After transfection, cells were then put back in culture in a T25 for 2 days, passage once in a T75 and culture for two more days. Cells were labeled using Halo-ligand-JF646 (provided by L. Lavis) at 100 nM in media, incubated for 30 min, washed three times in PBS, incubated three times in fresh media for 20 min with PBS washes between incubations. Cells were dissociated using Accutase (Invitrogen), washed twice in medium and resuspended in sorting buffer. Then, 1,000 positive cells were sorted on a FACSAria fusion. Cells were then put back in culture in a 10 cm petri dish coated with gelatin for 7 to 10 days before picking clones.

#### Clonal expansion

Single clonal colonies were manually picked under an EVOS cell imaging system, incubated in trypsin for 10 min and split 3/4–1/4 in two 96-well plates coated with gelatin and cultured for 2 days. The high-density plate was used for PCR genotyping and the low density plate for clone expansion.

### DNA and RNA pyrosequencing

DNA was extracted using DNeasy Blood and Tissue kit. RNA extraction was performed using the RNeasy kit and on-column DNase digestion (Qiagen). Reverse transcription was performed on 1 μg total RNA using SuperScript III (Life Technologies). To quantify allelic skewing, DNA or complementary DNA was amplified using the following biotinylated primers and subsequently sequenced using Q24 Pyromark (Qiagen).

### Western blot from nuclear extract

Nuclear extracts were prepared by collecting cells with trypsin, washing the pellet in PBS and resuspending the cells in ice-cold 10 ml buffer A (10 mM HEPES pH 7.9, 10 mM KCl, 1.5 mM MgCl_2_, 0.1% NP-40, c0mplete Mini Protease inhibitor EDTA free from Roche) and rotating for 10 min at 4 °C. Nuclei were centrifuged at 800 g for 10 min at 4 °C and resuspended in appropriate amount of RIPA buffer (50 mM Tris-HCl pH 8.0–8.5, 150 mM NaCl, 1% Triton X-100, 0.5% sodium deoxycholate, 0.1% SDS) containing cOmplete Mini Protease inhibitor (Roche), incubated for 20 min on ice and sonicated with a Bioruptor (four 5-s pulses). Lysates were then centrifuged for 30 min at 4 °C, and supernatants were kept. Protein concentration was determined using the Bradford (BioRad) assay. Samples were then boiled at 95 °C for 10 min in 3.2× LDS buffer (Thermo) containing 200 mM dithiothreitol. For RPB1, protein extracts were loaded on a 3–8% gradient gel in Tris-Acetate buffer. For RPB3, a 4–12% gel in MOPS buffer was used; as RPB3 and Lamin B have very similar size and cannot be revealed on the same membrane, extracts were loaded twice on the same gel. Transfer was performed on a 0.45-μm nitrocellulose membrane using a wet-transfer system, at 350 mA for 90 min at 4 °C. RPB1 membrane was cut in two so RPB1 and Lamin B (which came from the same loaded wells) were labeled separately; for RPB3 the membrane was cut in two to label one set of loaded wells for RPB3 and the other set of wells for Lamin B.

Rpb3 1:1,000 with second AB dilution 1:10,000

Lamin B1 1:3,000 with second AB dilution 1:10,000

Rpb1 1:500 with second AB dilution 1:5,000

### RNA FISH

#### Cell preparation

Cells were dissociated using Accutase (Invitrogen), washed twice in medium, and allowed to attach on poly-l-lysine (Sigma)-coated coverslips for 10 min. Cells were fixed with 3% paraformaldehyde in PBS for 10 min at room temperature, washed in PBS three times, and permeabilized with ice-cold permeabilization buffer (PBS, 0.5% Triton X-100, 2 mM vanadyl-ribonucleoside complex) for 4 min on ice, washed in 70% ethanol and stored in 70% ethanol at −20 °C or directly labeled.

#### Probes labeling and precipitation

The *Xist* probe was generated from a 19 kb genomic fragment covering most of *Xist* (2 kb of the promoter region plus exon 1 to mid-exon 6) (ref. ^46^). Huwe1 probe was an intron-spanning bacteria artificial chromosome (BAC) (clone RP24-157H12, available from BACPAC genomics at www.bacpacresources.org). Probes were prepared from phenol-chloroform extractions of the BAC or plasmid. Probes were labeled by nick translation (Abbott) using dUTP labeled with spectrum green (Abbott) for Huwe1 and Cy5 (Merck) for Xist. Labeled probes were precipitated in ethanol (3 μl of probes for plasmids and 5 μl of BAC, 100 μl of EtOH 100%, 1 μl of salmon sperm DNA, 0.7 μl of NaOAc 3 M pH 5.2 and for BAC probes adding 4 μl of Cot-1 repetitive DNA), washed in 70% ethanol, dried in a speedvac at room temperature, resuspended in formamide, denatured at 75 °C for 7 min, competed at 37 °C for 1 h for BAC probes with Cot-1 DNA and quenched on ice.

#### Hybridization

Samples were dehydrated in four baths of increasing ethanol concentration (80, 95 and 100% twice) and air-dried quickly. Probes were mixed in equal volume of hybridization buffer (7 μl of probes and 7 μl of buffer: 40% dextran sulfate, 2× SSC, BSA 2 mg ml^−1^, 10 mM vanadyl-ribonucleoside), spotted on cells and hybridized at 37 °C overnight. The next day, coverslips were washed three times for 7 min with 50% formamide in 2× SSC at 42 °C, and three times for 7 min with 2× SSC. DAPI (0.2 mg ml^−1^) was added to the last wash and coverslips were mounted with ProLong Diamond Antifade Mountant (Invitrogen).

#### Microscopy

RNA–FISH were imaged on a OLYMPUS SpinSR10 spinning disk microscope equipped with a Yokogawa CSU-W1 unit, a UPLSAPO ×100 S objective (NA 1,35, silicone oil) and using the SoRa disk (without additional magnification lens). 3D images were acquired with xx between stacks. For counting, Stacks were flattened into two dimensions by max projection, and cells with a *Xist* RNA cloud and/or *Huwe1* nascent RNA foci were counted manually.

### FCS–CI

#### Cell preparation

Cells were split at 50,000 cells per cm^2^ in ibidi eight-well chamber slides with glass bottom, coated with fibronectin. The chamber slides always contained one empty well for measurement on pure dye in solution, one well of ‘negative control’ cells (no Halo tag) and two wells of ‘free diffusing control’ Halo-NLS cells. Then, 24 h after splitting, cells were induced with doxycycline (2 μg ml^−^^1^). After 24 h of induction cells were labeled using Halo-ligand-JFX549 (provided by L. Lavis) in media containing doxycycline, incubated for 30 min, washed three times in PBS and incubated three times in fresh media (with doxycycline) for 20 min with PBS washes between incubations. For RPB1- and RPB3-Halo labeling was performed using ligand at 100 nM; for Halo-NLS, as the piggyBac transgene was expressed at a much higher level, one well was labeled with 5 nM and one with 2 nM, to allow a large range of fluorescence intensities for the calibration. ‘Negative control’ cells were labeled with 100 nM. Media (with doxycycline) was finally changed and pure AF568 dye in solution (5 nM) was added in the free well of the chamber before imaging.

#### Microscopy: FCS

FCS measurement and 3D imaging were performed on a Zeiss LSM880 microscope using a C-Apochromat Zeiss UV-visible-IR ×40/1.2-NA objective and operated with ZEN Blue software, equipped with an incubator chamber controlled at 37 °C and 8% CO_2_. FCS measurements were automated using the macro FCSRunner. The power of the 561 laser was set to 0.01 for all FCS measurements. For pure AF568 dye, two consecutive measurements of 10 s were performed for five points per field of view, for at least four fields of view per experiment. For all measurements in cells, a single measurement of 30 s was performed for a single point per cell, followed by one acquisition for the whole field of view, single stack at the same *z* position as the FCS measurement, with the same laser power and the following parameters: ×8 zoom and 128 × 128 pixels per field of view (resulting in a pixel size of 0.0991362 μm in *x*/*y*). All control measurements (Dye, free diffusing control and negative control) were performed each day for each individual experiment. Stage leveling was done manually based on the coverslip reflection, and re-done for each individual well of the chamber slide.

#### Microscopy: 3D acquisitions

3D acquisitions were performed on the same system as the FCS, following FCS acquisitions on the same day. Images were taken with the same parameters as FCS snapshots and 0.48 μm between *z* stacks.

#### FCS data processing

Background average signal in negative control cells was calculated using FCSFitM. FCS measurements were processed using FluctuationAnalyzer. All parameters were kept at default value except the following:

- Step Modify and correlate: ‘Base freq’: 1,000,000 (dye measurement) or 100,000 (cell)
- Step Intensity correction: ‘Base freq’: 1,000,000 (dye measurement) or 100,000 (cell)
  ‘Offset’: 0 (Dye) or the average intensity from negative cells (cell)
- Step Fit correlations: all fitting were performed using the model ‘two-component anomalous diffusion with triplet-like blinking’ with weighted fit, two runs of optimization and initial guess. For free dye and freely diffusing control Halo-NLS, the fraction of the first component was fixed to one resulting in practice in a one-component model. For Dye, the fitting was performed only on lag times from 0 to 10,239 μs to avoid overfitting the flat tail of the autocorrelation function.

#### Confocal volume estimation

The confocal volume was calculated based on FCS measurements on AF568 in solution based on the following equation:

1 $${V}_{\mathrm{conf}}={\pi }^{3/2}\kappa {w}_{0}^{3}$$

where *V*_conf_ is the effective confocal volume, *k* is the ratio of axial to lateral radius of this volume (estimated from the autocorrelation fitting) and *w*_0_ is the lateral radius of the confocal volume, which can be calculated following the following equation:

2 $${w}_{0}=2\times {({D}_{\mathrm{dye}}{\tau }_{\mathrm{dye}})}^{1/2}$$

where *D*_dye_ is the diffusion coefficient of the dye in solution (previously estimated to be *D*_AF568_ = 521.46 μm^2^ s^−1^ at 37 °C, ref. ^25^), *τ*_dye_ the diffusion time of the dye (estimated from the autocorrelation fitting) and *w*_0_ is the lateral radius of the confocal volume.

The average confocal volume was calculated based on all the dye measurements for each individual experiment separately.

#### Diffusion coefficient estimation

diffusion coefficients were calculated based on equation (2):

3 $${D}_{\mathrm{protein}}={({w}_{0}/2)}^{2}/{\tau }_{\mathrm{protein}}$$

where *D*_protein_ is the diffusion coefficient of the protein, *w*_0_ the lateral radius of the confocal volume estimated in the previous step based on dye measurements and *τ*_protein_ the diffusion time of the protein estimated from the autocorrelation fitting. Diffusion coefficients were calculated for each population from the fitting, the first one being the one with the highest diffusion coefficient (corresponding to the free fraction, Extended Data Fig. 5d).

#### FCS calibration

Calibration of pixel fluorescence intensity into concentration using paired two-dimensional (2D) imaging and FCS measurements was performed using a KNIME pipeline available on gitlab at https://git.embl.de/grp-almf/FCSpipelineEMBL_KNIME.

After that, paired 2D images and FCS measurement (analyzed using FluctuationAnalyzer as described above) are loaded, and the fluorescence intensity in the 2D image at the coordinates of the FCS point measurement is extracted. The fluorescence background is calculated as the average of fluorescence intensities at FCS points in negative control cells (not expressing any Halo tag), and this background is subtracted from the fluorescence intensity measurements for RPB1-Halo, RPB3-Halo and Halo-NLS. A linear trend between FCS measured concentrations and background corrected intensities is then fitted using least square regression:

4 $${C}_{\mathrm{FCS}}=a\times ({I}_{\mathrm{pixel}}-{I}_{\mathrm{background}})+b$$

where *C*_Fcs_ is the FCS measured concentration, *I*_pixel_ is the fluorescence intensity at the corresponding pixel on the 2D image and *I*_background_ is the background intensity.

This calibration is then used to convert pixel fluorescence intensities in 3D images into concentration:

5 $${C}_{\mathrm{voxel}}=a\times ({I}_{\mathrm{voxel}}-{I}_{\mathrm{background}})+b$$

where *C*_voxel_ is the inferred concentration per voxel in 3D images, *I*_voxel_ is the original fluorescence intensity per voxel for RPB1-/RPB3-Halo in 3D images, *I*_background_ is the background intensity and *a* and *b* are the parameters calculated in equation (4).

#### 3D image segmentation

Segmentation of nuclei, *Xist* territory, nucleoli and nucleoplasm were performed using ilastik^24^, first classifying pixels into different categories using the autocontext pixel classification function, and then segmenting the image based on the classifications. Different models were trained for the different regions: for nuclei, one model was trained using both the *Xist*-BglG-GFP and RBP1/3-Halo channels, with two annotations: background (between nuclei) and nuclei. For *Xist* territories, one model was trained using only the *Xist*-BglG-GFP channel; the RBP1/3-Halo was not used to not bias the segmentation of *Xist* territories based on the RPB1/3 intensities. Three annotations were used: background, *Xist* territory and the rest of the nucleus. Only the Xist territory classification was used from this model.

For nucleoli and nucleoplasm, a model was trained using both the *Xist*-BglG-GFP and RBP1/3-Halo channels, using four annotations: background, *Xist* territory (annotated as a high level of *Xist*-BglG-GFP), nucleoli (annotated as low level of RBP1/3 but no *Xist*-BglG-GFP) and nucleoplasm (rest of the nucleus). The annotation of *Xist* territory was done to avoid annotating those regions as nucleoli, as they both display lower levels of RPB1/3 and are frequently spatially close; however, the *Xist* territory classification from this model was not used in later analysis.

These classifications annotations were then used as input for the segmentation function (also done independently for each model).

Finally, the resulting segmentation of nuclei, *Xist* territory, nucleoli and nucleoplasm were exported in TIF format. The final segmentation was defined as follows: *Xist* territory, pixels belonging to ilastik segmentation of nucleus and *Xist* territory; nucleoli, pixels belonging to ilastik segmentation of nucleus and nucleoli but NOT *Xist* territory and nucleoplasm, pixels belonging to ilastik segmentation of nucleus and nucleoplasm but NOT *Xist* territory.

All ilastik models and corresponding files are available on github at https://git.embl.de/scollomb/collombet_et_al_rnapii_xist_compartment/-/tree/master/FCSCI/ilastik and all codes for FCS–CI data analysis are available on github at https://git.embl.de/scollomb/collombet_et_al_rnapii_xist_compartment/-/tree/master/FCSCI.

### SPT

#### Cell labeling

Cells were split at 50,000 cells per cm^2^ in 35 mm glass bottom dish (Mattek), coated with fibronectin. Then, 24 h after splitting, cells were induced with doxycycline (2 μg ml^−1^). After 24 h of induction, cells were labeled using Halo-ligand-PhotoActivable-JF646 (provided by L. Lavis) at 50 nM in media containing doxycycline, incubated for 30 min, washed three times in PBS and incubated four times in fresh media (with doxycycline) for 30 min with PBS washes between incubations. Media (with doxycycline) was finally changed before imaging.

#### Microscopy

Two-dimensional single particle tracking by photo-activated localization microscopy (2D SPT-PALM) was performed as previously described^26,27^ on a custom-built Nikon TI microscope (Nikon Instruments Inc.) equipped with a ×100/NA 1.49 oil-immersion TIRF objective (Nikon apochromat CFI Apo TIRF ×100 Oil), EM-CCD camera (Andor, iXon Ultra 897; frame-transfer mode; vertical shift speed 0.9 μs; −70 °C), a perfect focusing system to correct for axial drift and motorized laser illumination (Ti-TIRF, Nikon). A ×1.6 magnification lens was added in the light path allowing sampling at the objective Nyquist resolution, and resulting in a pixel size of 106 nm. The incubation chamber maintained a humidified 37 °C atmosphere with 5% CO_2_ and the objective was also heated to 37 °C. Lasers were modulated by an acousto-optic Tunable Filter (AA Opto-Electronic, France, AOTFnC-VIS-TN) and triggered with the camera through-the-lens exposure output signal. The microscope, cameras and hardware were controlled through NIS-Elements software (Nikon). The camera exposure time was set to 5 ms, the excitation with 633 nm laser (100% laser power) to 1 ms and the photoactivation with 405 nm laser synchronized with the off time of the camera (0.477 ms between frames). The intensity of the 405 laser was adapted manually between 2 and 10% during the acquisition to optimize photoactivation to obtain enough tracking per experiment while remaining sparse enough to track single molecules accurately. Per cell, 30,000 frames were acquired. Snapshots of Xist-BglG-GFP were taken before and after the SPT with the 488 nm laser (200 ms exposure).

#### Localization and tracking

Localization and tracking were performed using the pyspaz program (https://github.com/alecheckert/pyspaz). Localization was performed using the function localize detect-and-localize-file with the following parameters: -s 1 -t 20, and all other parameters as default. Tracking was performed using the function track track-locs with the following parameters: --algorithm_type conservative --pixel_size_um 0.106 -e 3 -dm 10 -db 0.1 -f 5.477 -b 0 and all other parameters as default.

#### Segmentation

For each acquisition, the two Xist-Bgl-GFP snapshots (before and after SPT) were combined as one multidimensional TIF file using a custom python script. These snapshots were used for segmentation of the *Xist* compartment and nucleoplasm using ilastik. The autocontext mode was first used to create probability maps, annotating pixels as *Xist* territory (high *Xist*-BglG-GFP signal), nucleoplasm (low *Xist*-BglG-GFP signal) and ‘background’ (between nuclei). The Tracking mode was then used with the probability maps as input to automatically segment and annotate nuclei and *Xist* territory (one model built to track *Xist* territory, one model to track nuclei). To segment nucleoli, the density of single-particle localizations from the 10,000 first and last frames was calculated (a large number of frames is required to capture enough particles and avoid artificial ‘empty regions’). The SPT densities and Xist-Bgl-GFP snapshots were combined into one multichannel image, and ilastik was used to segment nucleoli, Xi and nucleoplasm using the same strategy as for Xist compartment segmentation. All ilastik models and corresponding files are available on github at https://git.embl.de/scollomb/collombet_et_al_rnapii_xist_compartment/-/tree/master/SPT/ilastik.

#### Trajectories assignment to subcompartments

Assignment of trajectories to nuclei, nucleoplasm or XC was performed using a custom python script interpolateMaskAndAssignTrajectories.py available on our github at https://git.embl.de/scollomb/collombet_et_al_rnapii_xist_compartment/-/blob/master/SPT/interpolateMaskAndAssignTrajectories.py. We used the following parameters: --olap_fracMin 0 --olap_fracMax 1 --pixel_subsampling_factor 1. For trajectories inside XC were defined as those for which at least one localization was found inside the XC mask (--olap_rule any) and trajectories outside XC as those for which no localization was found inside the mask (--olap_rule none).

#### Trajectories entering XC and control regions

Control regions were created using a custom python script interpolateMaskAndAssignTrajectories_moveMask_ROA.py available on our github at https://git.embl.de/scollomb/collombet_et_al_rnapii_xist_compartment/-/blob/master/SPT/interpolateMaskAndAssignTrajectories_moveMask_ROA.py. In summary, this script finds control XC regions by randomly shifting and rotating the XC segmentation mask, while controlling that the shifted mask remains inside the nucleus, does not overlap with a previous mask and does not overlap with nucleoli. It takes as input the mask of XC, nuclei and nucleoli, randomly shift and rotate the XC mask and evaluate whether the new mask respects four rules: (1) the shifted mask is entirely inside the nucleus (--maxMaskFracOutsideROE 0.0), (2) its distance to the nuclear periphery is not different from the original mask by more than 50% (--minMaskDistRoeDifFrac -0.5 --maxMaskDistRoeDifFrac 0.5), (3) it does not overlap nucleoli by more than 1% of its size (--maxOlapROA 0.01) and (4) it does not overlap the original mask or a previously valid shifted mask by more than 10% of their respective size (--maxMasksOlap 0.1).

If the shifted mask respects these rules, it is added to the list of control regions. This operation is repeated until ten control regions are found or 500,000 iterations are performed (we did not see the number of control regions per cell increase with higher number of iterations).

#### Distribution of diffusion coefficient

The distribution of diffusion coefficient for RPB1/3 inside/outside *Xist* compartment was estimated using spagl^30^ available at https://github.com/alecheckert/spagl, using the fss_plot function with default parameters (dz = 0.7, pixel_size_um = 0.106, frame_interval_sec = 0.005477).

#### Bound/free fraction estimation

To estimate the bound and free fractions, a two-component model was fitted to the distribution of jumps using *S*potOn^26^. A custom version of the python implementation of SpotOn was adapted to run on python 3, which can be found on our github at https://git.embl.de/scollomb/collombet_et_al_rnapii_xist_compartment/-/tree/master/SPT/Spot-On-cli. The function fit-and-plot-2states was used with the following parameters: --time_between_frames 0.5477 --gaps_allowed 0 --localisation_error 0.028 --weight_delta_t True --model_fit CDF --max_jump_length 2 --max_jumps_per_traj 3 --max_delta_t 6 --diffusion_bound_range 0,0.02 --diffusion_free_range 0.1,20.

#### Jumps angles

The distribution of jump angles was calculated using the function plot-jumps-angle-circular from our implementation of SpotOn (github link) with the following parameters: --gaps_allowed 0 --min_1dt_jump_length 0.2 --max_1dt_jump_length 3 --max_jumps_per_traj 100 --delta_t 1 --bin_width 10.

#### Bootstrap analysis

Bootstrap was performed using the function subsample-trajs from our implementation of SpotOn. All codes for SPT analysis are available on github at https://git.embl.de/scollomb/collombet_et_al_rnapii_xist_compartment/-/tree/master/SPT.

### FRAP

#### Cell labeling

Cells were prepared the same way as for FCS–CI: split at 50,000 cells per cm^2^ in ibidi eight-well chamber slides with glass bottom and coated with fibronectin. Then 24 h after splitting, cells were induced with doxycycline (2 μg ml^−1^). After 24 h of induction, cells were labeled using Halo-ligand-JFX549 (provided by L. Lavis) at 100 nM in media containing doxycycline, incubated for 30 min, washed three times in PBS and incubated three times in fresh media (with doxycycline) for 20 min with PBS washes between incubations. Media (with doxycycline) was finally changed before imaging.

#### RNAPII inhibition

Before imaging, media were supplemented with DRB at 500 μM or Flavopiridol 10 μM for 2–3 h and imaged in the following 2 h. High concentrations and treatment time were used to ensure complete inhibition of RNAPII elongation (for reference values, see ref. ^34^). The total time of treatment (2 h to 5 h maximum) was set as the minimal time to reach full inhibition but before affecting cell viability. Of note, we observed that cells remain viable up to 7–8 h of treatment, after which massive cell death was observed. We therefore performed FRAP in the 2–5 h window where inhibition is complete and cell viability is not affected.

#### Microscopy

FRAP was performed on the same microscope setup as FCS–CI using the same objective. Imaging was done with a ×18 zoom and an optimal frame size of 104 pixels, a speed of 18 corresponding to a dwelling time of 1.28 μs per pixel and a scan time of 31.95 ms per frame. A snapshot was first taken using both 488 channel and 561 channels to visualize the *Xist* territory. Regions of interest (ROI) were defined as circles of 10 pixels (to be always fully contained in the *Xist* territory) into the *Xist* territory, nucleoplasm and background (outside cells). FRAP acquisition was then performed only using the 561 channel to allow fast imaging with roughly 32 ms between frames. Photobleaching was performed after 80 frames on the *Xist* territory circle (or a second nucleoplasm region for bleaching control in the nucleoplasm), with a scanning speed of seven (corresponding to roughly 9 ms bleaching time) and a laser poxer of 60% (both parameters were manually optimized to allow more than 50% bleaching inside the defined circle while minimizing bleaching of the surrounding area).

#### Data analysis

To correct for bleaching and background, an exponential decay function was fitted to the background and nucleoplasm regions measurements.

The signal in the bleached ROI at all time points was then scaled to the prebleached signal, and the background was subtracted as follows:

6 $${\mathrm{Ic}}_{n}^{\mathrm{ROI}}=({I}_{n}^{\mathrm{ROI}}-{I}_{n}^{({\mathrm{bkg.exp}})})/(\langle {I}_{t0-{\mathrm{bleach}}}^{\mathrm{ROI}}\rangle -\langle {I}_{t0-{\mathrm{bleach}}}^{\mathrm{bkg.exp}}\rangle )$$

where ${\mathrm{Ic}}_{n}^{\mathrm{ROI}}$ is the background and prebleached scaled intensity in ROI at time *n*, ${I}_{n}^{\mathrm{ROI}}$ is the raw intensity in the ROI at time *n*, ${I}_{n}^{\mathrm{bkg.exp}}$ is the exponential fit of the background signal (outside cell) at time *n*, $\langle {I}_{t0-{\mathrm{bleach}}}^{\mathrm{ROI}}\rangle$ is the mean of signal in the ROI before bleaching time and $\langle {I}_{t0-{\mathrm{bleach}}}^{\mathrm{bkg.exp}}\rangle$ is the mean of the background fitted signal.

Bleaching was then corrected using the signal in the control region:

7 $${\mathrm{Icb}}_{n}^{\mathrm{ROI}}={\mathrm{Ic}}_{n}^{\mathrm{ROI}}/(({I}_{n}^{\mathrm{ROC.exp}}-{I}_{n}^{\mathrm{bkg.exp}})/(\langle {I}_{t0-{\mathrm{bleach}}}^{\mathrm{ROC.exp}}\rangle -\langle {I}_{t0-{\mathrm{bleach}}}^{\mathrm{bkg.exp}}\rangle ))$$

where ${\mathrm{Ic}}_{n}^{\mathrm{ROI}}$ is the corrected signal in ROI at time *n* calculated in ref. ^6^, ${I}_{n}^{\mathrm{ROC.exp}}$ is the exponentially fitted intensity in the control region (inside the nucleus, nonbleached) at time *n* and $\langle {I}_{t0-{\mathrm{bleach}}}^{\mathrm{ROC.exp}}\rangle$ the mean of exponentially fitted intensity in the control region before bleaching.

### Reporting summary

Further information on research design is available in the Nature Portfolio Reporting Summary linked to this article.

## Online content

Any methods, additional references, Nature Portfolio reporting summaries, source data, extended data, supplementary information, acknowledgements, peer review information; details of author contributions and competing interests; and statements of data and code availability are available at 10.1038/s41594-023-01008-5.

## Supplementary information

Reporting Summary

## Data Availability

Segmentation data for ilastik model training are available on github: https://git.embl.de/scollomb/collombet_et_al_rnapii_xist_compartment. All main data supporting the findings of this study are available within the article, Extended Data and Supplementary information. Source data are provided with this paper.

## Source data

Source data for Extended Data Fig. 1 Unprocessed western blots.

## Code availability

All the codes for FCS–CI and SPT analysis are available on github: https://git.embl.de/scollomb/collombet_et_al_rnapii_xist_compartment.
