# Supplementary material for: RNA polymerase II depletion from the inactive X chromosome territory is not mediated by physical compartmentalization
Source: Nat Struct Mol Biol. 2023 Jun 8;30(8):1216–23. doi: 10.1038/s41594-023-01008-5 (PMC10442225; doi:10.1038/s41594-023-01008-5)
Supplement: Supplementary file 1 — Reporting Summary [file 41594_2023_1008_MOESM1_ESM.pdf]

nature research

Corresponding author(s): Edith Heard

Last updated by author(s): Nov 9, 2021

Reporting Summary

Nature Research wishes to improve the reproducibility of the work that we publish. This form provides structure for consistency and transparency in reporting. For further information on Nature Research policies, see our Editorial Policies and the Editorial Policy Checklist.

Statistics

For all statistical analyses, confirm that the following items are present in the figure legend, table legend, main text, or Methods section.

n/a	Confirmed	
☐	☒	The exact sample size (*n*) for each experimental group/condition, given as a discrete number and unit of measurement
☐	☒	A statement on whether measurements were taken from distinct samples or whether the same sample was measured repeatedly
☐	☒	The statistical test(s) used AND whether they are one- or two-sided<br>*Only common tests should be described solely by name; describe more complex techniques in the Methods section.*
☐	☒	A description of all covariates tested
☐	☒	A description of any assumptions or corrections, such as tests of normality and adjustment for multiple comparisons
☐	☒	A full description of the statistical parameters including central tendency (e.g. means) or other basic estimates (e.g. regression coefficient) AND variation (e.g. standard deviation) or associated estimates of uncertainty (e.g. confidence intervals)
☐	☒	For null hypothesis testing, the test statistic (e.g. *F*, *t*, *r*) with confidence intervals, effect sizes, degrees of freedom and *P* value noted<br>*Give P values as exact values whenever suitable.*
☐	☒	For Bayesian analysis, information on the choice of priors and Markov chain Monte Carlo settings
☐	☒	For hierarchical and complex designs, identification of the appropriate level for tests and full reporting of outcomes
☐	☒	Estimates of effect sizes (e.g. Cohen's *d*, Pearson's *r*), indicating how they were calculated

Our web collection on statistics for biologists contains articles on many of the points above.

Software and code

Policy information about availability of computer code

Data collection	Confocal imaging was acquired using Zeiss Zen Blue software ; Single particle tracking were acquired using Nikon NIS-Elements software.
Data analysis	All analysis were performed in R 4.2 and python 3. Image segmentation wa performed using Ilastik 1.3.3.<br>The KNIME pipeline for FCS-CI analysis is available on github: https://git.embl.de/grp-almf/FCSpipelineEMBL_KNIME<br>The python library used for SPT analysis is available on github: https://github.com/alecheckert/pyspaz<br>The code for all analysis is available on github: https://git.embl.de/scollomb/collombet_et_al_rnapii_xist_compartment/-/tree/master

For manuscripts utilizing custom algorithms or software that are central to the research but not yet described in published literature, software must be made available to editors and reviewers. We strongly encourage code deposition in a community repository (e.g. GitHub). See the Nature Research guidelines for submitting code & software for further information.

Data

Policy information about availability of data

All manuscripts must include a data availability statement. This statement should provide the following information, where applicable:

- Accession codes, unique identifiers, or web links for publicly available datasets
- A list of figures that have associated raw data
- A description of any restrictions on data availability

All data for segmentation model training are available on github: https://git.embl.de/scollomb/collombet_et_al_rnapii_xist_compartment

Field-specific reporting

Please select the one below that is the best fit for your research. If you are not sure, read the appropriate sections before making your selection.

☒ Life sciences ☐ Behavioural & social sciences ☐ Ecological, evolutionary & environmental sciences

For a reference copy of the document with all sections, see nature.com/documents/nr-reporting-summary-flat.pdf

Life sciences study design

All studies must disclose on these points even when the disclosure is negative.

Sample size	Sample size were no predetermined. All experiments were reproduced at least 3 independent times on 3 different days. Single particle tracking experiment were reproduce to obtain a minimum of 3000 trajectories which was previously showed to be sufficient to provide robust estimation, as proven by our bootstrap analysis.
Data exclusions	no data were excluded.
Replication	All experiments were reproduced at least 3 independent times on 3 different days.
Randomization	no randomization was performed.
Blinding	no blinding was performed.

Reporting for specific materials, systems and methods

We require information from authors about some types of materials, experimental systems and methods used in many studies. Here, indicate whether each material, system or method listed is relevant to your study. If you are not sure if a list item applies to your research, read the appropriate section before selecting a response.

Materials & experimental systems

n/a	Involved in the study	
☐	☒	Antibodies
☐	☒	Eukaryotic cell lines
☒	☐	Palaeontology and archaeology
☒	☐	Animals and other organisms
☒	☐	Human research participants
☒	☐	Clinical data
☒	☐	Dual use research of concern

Methods

n/a	Involved in the study	
☒	☐	ChIP-seq
☒	☐	Flow cytometry
☒	☐	MRI-based neuroimaging

Antibodies

Antibodies used	Lamin B1, Abcam cat. #ab16048, WB, 1/3000<br>Rpb1, Abcam cat. #ab817, WB, 1/500<br>Rpb3, Abcam cat. #ab181193 WB, 1/1000
Validation	*Describe the validation of each primary antibody for the species and application, noting any validation statements on the manufacturer's website, relevant citations, antibody profiles in online databases, or data provided in the manuscript.*

Eukaryotic cell lines

Policy information about cell lines

Cell line source(s)	TX1072 (female mouse ESC line developed in the Heard lab, available on demand)
Authentication	RNA FISH and pyrosequencing was performed after each genome editing to exclude chromosomic abberations.
Mycoplasma contamination	All cell lines were frequently tested and are mycoplasma negative.
Commonly misidentified lines (See ICLAC register)	NA
