# Supplementary material for: RNA polymerase II depletion from the inactive X chromosome territory is not mediated by physical compartmentalization
Source: Nat Struct Mol Biol. 2023 Jun 8;30(8):1216–23. doi: 10.1038/s41594-023-01008-5 (PMC10442225; doi:10.1038/s41594-023-01008-5)

Source Data, for Extended Data Fig 1

RBP3
(first panel from top)

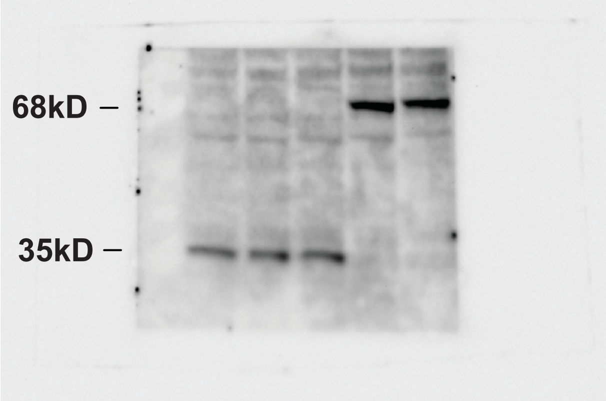

RBP1
(third panel from top)

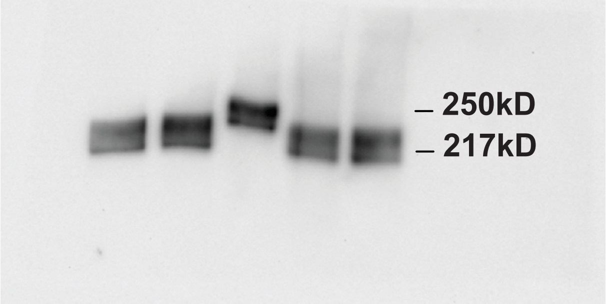

Lamin B control for RBP3
(second panel from top)

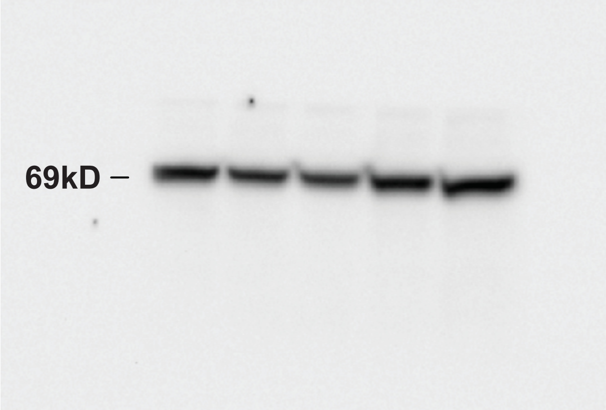

Lamin B control for RBP1
(fourth panel from top)

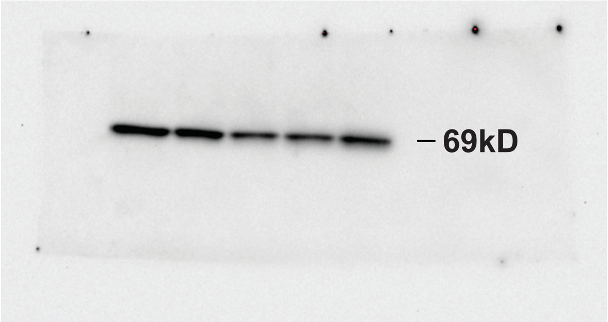

Supplement: Source data for Extended Data Fig. 1 — Unprocessed western blots. [file 41594_2023_1008_MOESM2_ESM.pdf]
